# The Technical Feasibility of Integrating Primary Eye Care Into Primary Health Care Systems in Nigeria: Protocol for a Mixed Methods Cross-Sectional Study

**DOI:** 10.2196/17263

**Published:** 2020-10-27

**Authors:** Ada Aghaji, Helen Burchett, Shaffa Hameed, Jayne Webster, Clare Gilbert

**Affiliations:** 1 Department of Ophthalmology College of Medicine Enugu Nigeria; 2 Department of Clinical Research London School of Hygiene & Tropical Medicine London United Kingdom; 3 Department of Public Health, Environments and Society London School of Hygiene & Tropical Medicine London United Kingdom; 4 Department of Disease Control London School of Hygiene & Tropical Medicine London United Kingdom

**Keywords:** primary eye care, primary health care, implementation, visual impairment, technical feasibility, feasibility study, health policy, Nigeria, World Health Organization Regional Office for Africa, WHO-AFRO

## Abstract

**Background:**

Approximately 90% of the 253 million blind or visually impaired people worldwide live in low- and middle-income countries. Lack of access to eye care is why most people remain or become blind. The World Health Organization Regional Office for Africa (WHO-AFRO) recently launched a primary eye care (PEC) package for sub-Saharan Africa—the WHO-AFRO PEC package—for integration into the health system at the primary health care (PHC) level. This has the potential to increase access to eye care, but feasibility studies are needed to determine the extent to which the health system has the capacity to deliver the package in PHC facilities.

**Objective:**

Our objective is to assess the technical feasibility of integrating the WHO-AFRO PEC package in PHC facilities in Nigeria.

**Methods:**

This study has several components, which include (1) a literature review of PEC in sub-Saharan Africa, (2) a Delphi exercise to reach consensus among experts regarding the technical complexity of the WHO-AFRO PEC package and the capacities needed to deliver it in PHC facilities, (3) development of PEC technical capacity assessment tools, and (4) data collection, including facility surveys and semistructured interviews with PHC staff and their supervisors and village health workers to determine the capacities available to deliver PEC in PHC facilities. Analysis will identify opportunities and the capacity gaps that need to be addressed to deliver PEC.

**Results:**

Consensus was reached among experts regarding the technical complexity of the WHO-AFRO PEC package and the capacities needed to deliver it as part of PHC. Quantitative tools (ie, structured questionnaires, in-depth interviews, and observation checklists) and topic guides based on agreed-upon technical capacities have been developed and relevant stakeholders have been identified. Surveys in 48 PHC facilities and interviews with health professionals and supervisors have been undertaken. Capacity gaps are being analyzed.

**Conclusions:**

This study will determine the capacity of PHC centers to deliver the WHO-AFRO PEC package as an integral part of the health system in Nigeria, with identification of capacity gaps. Although capacity assessments have to be context specific, the tools and findings will assist policy makers and health planners in Nigeria and similar settings, who are considering implementing the package, in making informed choices.

**International Registered Report Identifier (IRRID):**

DERR1-10.2196/17263

## Introduction

Approximately 253 million people are blind or visually impaired worldwide, 90% of whom live in low- and middle-income countries (LMICs) [1]. In Nigeria, about 4.25 million adults are blind or visually impaired and over 80% of the blindness is due to avoidable causes [2,3]. Lack of access to eye care services is one of the reasons why people remain or become blind [4]. Cataracts are the most common cause of blindness in Nigeria [2], and high-quality cataract surgery should be accessible and affordable for all. However, in the Nigeria National Blindness and Visual Impairment Survey, almost half of all eyes that had undergone a procedure for cataract treatment had undergone couching—a traditional procedure to treat cataracts—often with poor visual outcomes. Glaucoma, which causes irreversible visual loss, was the second-most common cause of blindness [2]. Although early treatment can prevent or slow progression of the disease, in Nigeria people with glaucoma present very late to eye care services, often already blind in one or both eyes.

Other blinding-eye conditions in Nigeria include uncorrected refractive error [5], trachoma, and diabetic retinopathy. Presbyopia, the age-related decline in near vision, affects an estimated 20 million adults in Nigeria [6] and can lead to considerable productivity losses if uncorrected. Although blindness in children is rarer than in adults, many of the blinding conditions in LMICs, such as measles infection and vitamin A deficiency, can be prevented at the primary level [7,8].

Other eye conditions that cause ocular morbidity for which access to eye care is needed include dry, irritable eyes and allergic and infective conjunctivitis [9]. There is, therefore, a need for LMICs to provide universal access to eye care, not just for blinding conditions but also for conditions causing troublesome symptoms. Approximately 25% of Nigerians have ocular conditions [9]; with a population of 200 million, this means that approximately 50 million Nigerians are in need of eye care.

In LMICs, most eye care is delivered in secondary- and tertiary-level facilities, which are mainly located in urban areas. This leads to inequity in access, higher costs for patients and providers [10], and the patronage of other sources of care (eg, informal drugs sellers, traditional and spiritual healers, and couchers), which may exacerbate the visual loss through harmful practices or delayed access to appropriate treatment [9,11]. Over 35% of Nigerians with ocular problems consult an informal drug seller as a first option, primarily due to a lack of access to eye care services [9].

One way to improve access to eye care in LMICs is to integrate eye care into primary health care (PHC) [10], which is advocated by the World Health Organization (WHO) in their report Universal Eye Health: Global Action Plan 2014-2019 [12]. Primary eye care (PEC) entails the following elements: health protection, health promotion, specific preventive measures, detection and treatment of common eye conditions, detection and referral of more complex conditions, and record keeping. The health promotion elements can be delivered in the community as well as in facilities, while the other components principally take place in PHC facilities.

However, delivering PEC can have challenges; these include low PEC skill levels [13], inadequate supervision [14], and inadequate equipment and supplies [15]. A review of the literature on PEC in sub-Saharan Africa showed that there has been no consensus on the scope of PEC nor guidelines on the technical skills PHC workers require to implement eye care; this has resulted in deficient training and inadequate supervision [16]. To encourage uniformity of the scope of PEC in sub-Saharan Africa, the WHO Regional Office for Africa (WHO-AFRO) recently launched a package of evidence-based interventions for PEC: the WHO-AFRO PEC package [17]. The package can be subdivided into two broad elements: health promotion and facility-based eye care. The latter comprises five evidence-based algorithms and protocols on how to measure distance and near visual acuity, administer eye medication, remove foreign bodies, apply an eye patch, document findings, and refer and counsel patients. The purpose of the package is to strengthen the capacity of PHC workers in sub-Saharan Africa to manage patients with eye conditions [17] and widen access to eye care [18]. The package has been pilot-tested in Rwanda and Kenya [18].

**Figure 1:**
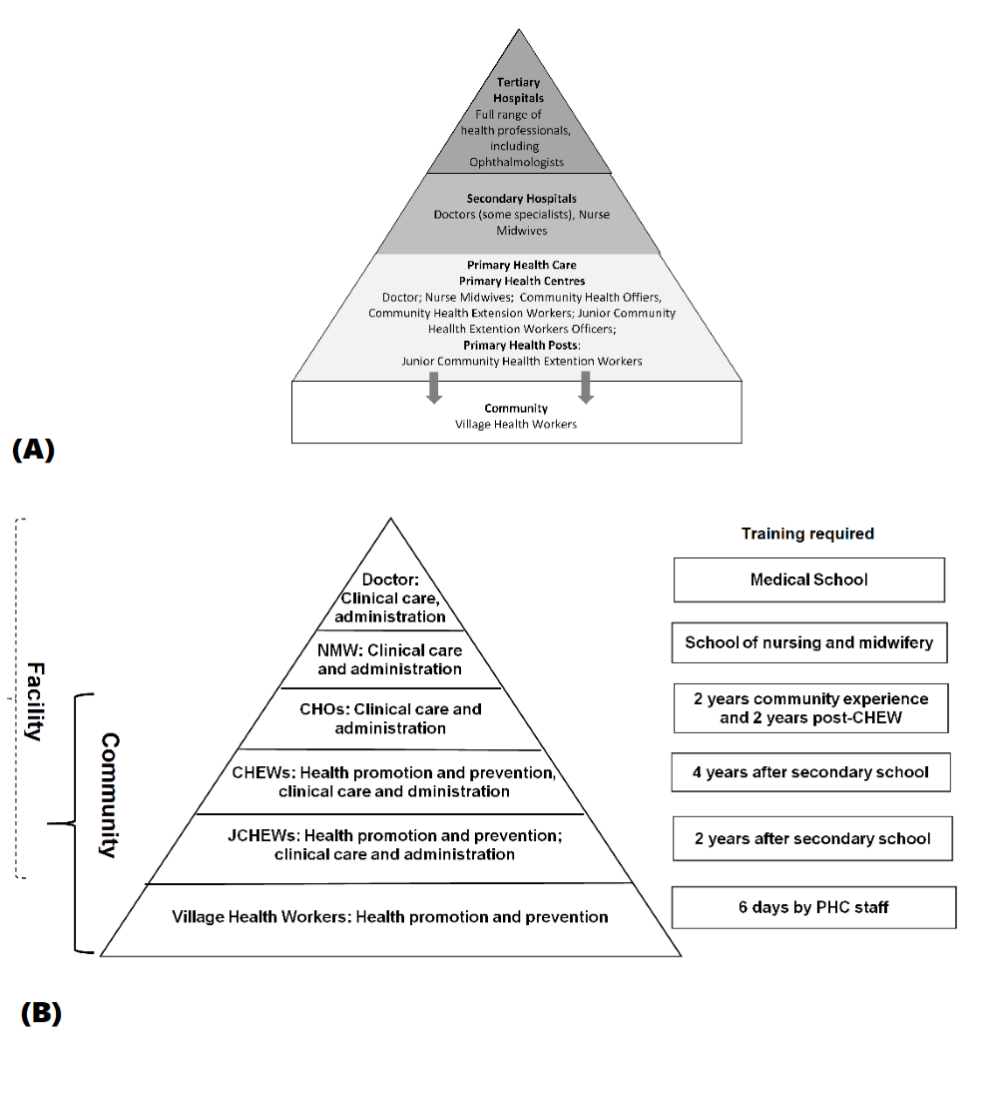
Human resources for health in Nigeria (A) across the tiers of the health system and (B) at primary health care (PHC) facilities. CHEW: community health extension worker; CHO: community health officer; JCHEW: junior community health extension worker; NMW: nurse midwife.

In Nigeria, the health system has three tiers of service delivery—primary, secondary, and tertiary (see Figure 1, A)—staffed by appropriate cadres. The PHC system provides basic services and is often the first point of contact with the health system and the only source of health care for the majority of Nigerians in rural and remote communities [19]. PHC is delivered in health centers and smaller units called health posts. PHC staff comprise junior community health extension workers, community health extension workers (CHEWs), community health officers, and nurse midwives (see Figure 1, B).

Challenges of delivering PHC in Nigeria include, but are not limited to, shortage of health workers and absenteeism [20], infrastructural decay and poor funding [21], a dearth of basic equipment [22], and lack of trust in the system by the community [23]. Attempts have been made by nongovernmental organizations to implement PEC in some parts of Nigeria by training a limited number of staff using their own curricula and providing basic equipment. However, these initiatives were only scaled up with financial support in one state, so they were not scalable nor sustainable [24]. Nevertheless, to deliver an effective and sustainable intervention, it is important that feasibility studies are conducted in each implementation setting. Feasibility research can help identify the opportunities and challenges in implementing a new health initiative, including PEC, which can only be as efficient as the PHC system into which it is built [16].

Feasibility is a complex construct [25], which has been defined in different ways. For example, Snowden et al define feasibility as encompassing the following domains: political, cultural, or community acceptability as well as technical, cost, and legal feasibility [26]. This study focuses on technical feasibility, which comprises the technical complexity of an intervention and the technical capacities needed to deliver it [27]. To our knowledge, no technical feasibility study in relation to PEC in sub-Saharan Africa has been undertaken. Identifying the challenges, opportunities, and gaps in the technical capacities required will provide information for policy makers to make informed decisions about how the health system needs to be strengthened to deliver PEC as an integral component of PHC. The research is timely, as PHC reforms are currently underway in Nigeria, which include national policies to train primary-level staff and to provide essential drugs and consumables under the umbrella of Universal Health Coverage. These initiatives provide real opportunities to integrate PEC into PHC [24]. The overarching aim of this study is to determine the technical feasibility of implementing the WHO-AFRO PEC package into PHC facilities in Nigeria; in this paper, we describe, in detail, the methods to achieve this.

## Methods

### Overview

**Figure 2:**
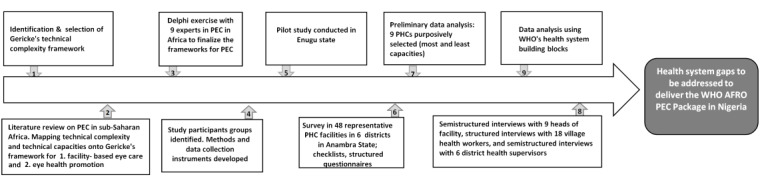
Flowchart of the study. PEC: primary eye care; PHC: primary health care; WHO-AFRO: World Health Organization Regional Office for Africa.

This study has several components, including a literature review on PEC in sub-Saharan Africa; a Delphi exercise to reach consensus among experts regarding the technical complexity of the WHO-AFRO PEC package and the capacities needed to deliver it in PHC facilities; development of PEC technical capacity assessment tools; and data collection, including facility surveys and semistructured interviews with PHC staff and their supervisors and village health workers (VHWs) to determine the capacities available to deliver PEC in PHC facilities (see Figure 2). Analysis will identify opportunities and the capacity gaps that need to be addressed to deliver PEC.

### Components of the Study

#### Identification and Selection of Theoretical Framework

**Figure 3:**
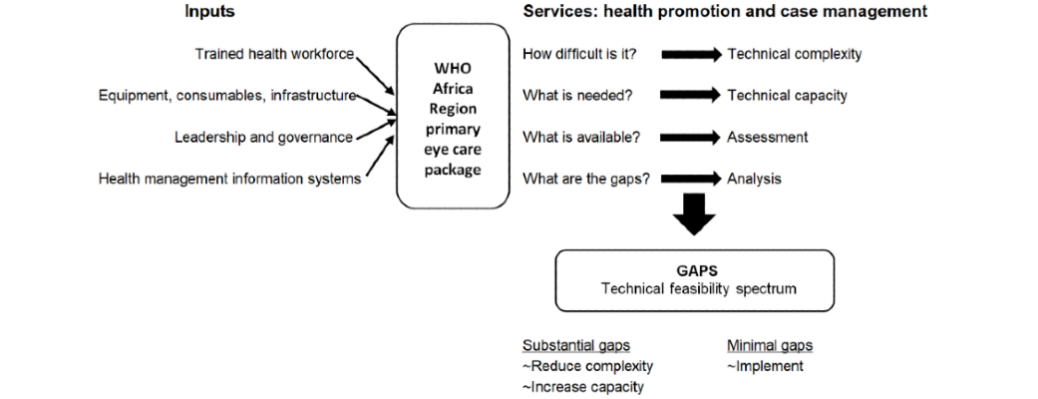
Theoretical framework for technical feasibility assessment derived from Gericke et al [27]. WHO: World Health Organization.

There are only a few analytical tools to assess the technical complexity of an intervention, one of which was proposed by Gericke et al [27]. This framework comprises four domains: (1) intervention characteristics, (2) delivery characteristics, (3) government capacity requirements, and (4) usage characteristics with criteria for each (see Table 1 [27]). A theoretical framework for assessing the technical feasibility of implementing the WHO-AFRO PEC package in PHC facilities in Nigeria has been designed by the investigators (see Figure 3 [27]), which builds on Gericke’s framework of technical complexity [27]. This involves assessing the complexity of each component of the intervention and, from this, extrapolating to the technical capacities required to implement it. Analysis of the data collected will reveal the gaps that need to be addressed, which may be minimal or substantial. The size and nature of the gaps will determine the feasibility of implementing the intervention.

The Delphi method is a scientific, multistage approach to achieve consensus from combined expert opinion through a series of structured questionnaires completed anonymously. Advantages of the Delphi approach include anonymity and the achievement of consensus where definitive evidence is lacking [28,29].

A two-round Delphi exercise was used to build consensus on, first, the technical complexity of the WHO-AFRO PEC package and, second, the technical capacities required to implement it. The study was conducted over 5 months in 2018.

**Table 1:** Gericke et al’s framework to assess the technical complexity of health interventions [27].

Category		Criteria
**Intervention characteristics**		
	Basic product design	Stability Standardizability Safety profile Ease of storage Ease of transport
	Supplies	Need for regular supplies
	Equipment	High-technology equipment and infrastructure needed A number of different types of equipment needed Maintenance needed
**Delivery characteristics**		
	Facilities	Retail sector Outreach services First-level care Hospital care
	Human resources	Skill level required for service provision Skill level required for staff supervision Intensity of professional services in terms of frequency or duration Management and planning requirements
	Communication and transport	Dependence of delivery on communication and transport infrastructure
**Government capacity requirements**		
	Regulation and legislation	Need for regulation Need for monitoring regulatory measures and enforcement of regulation
	Management systems	Need for sophisticated management systems
	Collaborative action	Need for intersectoral action within government Need for partnership between government and external funding agencies
**Usage characteristics**		
	Ease of use	Need for information and education Need for supervision
	Pre-existing demand	Need for promotion
	Black market risk	Need to prevent resale and counterfeiting

#### Literature Review of PEC in Sub-Saharan Africa and Mapping Onto the Appropriate Segment of Gericke’s Framework to Form the Delphi Questionnaire

The WHO-AFRO PEC package was divided into two components: eye health prevention and promotion and facility-based case management. Gericke’s framework was used to develop two questionnaires, one for health promotion and the other for facility-based care, which were entered into a Microsoft Excel 2016 spreadsheet.

In order to populate the two questionnaires, a literature search on PEC in sub-Saharan Africa was undertaken based on literature published in PubMed up to April 2018. Search terms included “primary eye care” and “sub-Saharan Africa.” The bibliographies of the two most recent published reviews on PEC in sub-Saharan Africa were also reviewed [16,30]. A total of 173 articles were retrieved. Articles that were not related to PEC in sub-Saharan Africa were excluded, leaving 51 articles for inclusion, including 2 randomized trials.

Further implementation characteristics were identified by two of the authors (CG and AA) who have more than 40 years’ combined experience in eye care in sub-Saharan Africa. These two sources yielded a list of key criteria for the technical complexity of PEC, which were used to populate the Delphi questionnaires. A 4-point Likert scale, ranging from 1 (strongly agree) to 4 (strongly disagree), was applied to each statement. The Delphi questionnaires were reviewed by an expert in international eye health (CG), a health interventions expert (HB), and a statistician (David MacCleod, London School of Hygiene & Tropical Medicine).

#### The Delphi Exercise

##### Selection of Experts for the Delphi Exercise

The main eligibility criteria for the Delphi panel included being an eye care professional with long-standing experience in community eye care in sub-Saharan Africa, preferably for a minimum of 10 years and who is still professionally active, and having experience in eye health policy. Panel members were selected by a modified, exponential, snowball-sampling method where an initial participant provides multiple referrals [31]. Each new referral was vetted and included in the study if the eligibility criteria were met.

##### Delphi Rounds

A total of 12 panel members were contacted by email and telephone, 9 of whom confirmed their willingness to take part. All 9 completed both rounds of the Delphi exercise.

For the first round, panel members were sent the following documents: the methods to be used during the Delphi exercise, an explanation of Gericke’s framework of technical complexity, and the first pair of questionnaires on the technical complexities of PEC. Participants were invited to state their level of agreement with each statement by ticking the appropriate level in the Likert scale. A comments box was included beside each statement for comments or suggestions.

Once all the questionnaires had been received, they were analyzed for consensus, defined as at least 70% agreement on each statement in the upper-50th percentile (Likert scale scores of 1 and 2). Where consensus was reached, the statements were adopted. Statements where consensus was not reached were modified based on the suggestions and comments and were incorporated into the second round, as were newly identified statements.

For the second round, each of the agreed-upon statements on technical complexity were modified to reflect the technical capacities required for delivery, and Likert scales were added. Panel members were sent the questionnaires on technical capacities, which included the comments and suggestions of participants from the first round. Only statements that achieved consensus, as defined as above, were included in the final document. Any minority views (<70% consensus) were not adopted but were documented.

#### Development of PEC Technical Capacity Assessment Tools and Selection of Participant Groups

##### Overview

The technical capacities derived from the Delphi exercise were mapped onto the WHO’s health systems framework, which comprises the health workforce, leadership and governance, financing, health management information systems, equipment, technology and infrastructure, and service delivery [32]. After reviewing the capacities needed, the optimal method of assessment was determined (ie, document review; structured questionnaires; observational checklists; in-depth interviews, using a structured topic guide; and the relevant participant groups: VHWs; PHC staff, including facility heads; CHEWs; and district supervisors) (see Table 2). Mixed methods were used to ensure a comprehensive understanding and to triangulate the data [33]. The instruments were developed in English and interviews were conducted in English, except for the VHW questionnaire, which was translated into Igbo and back-translated into English to ensure it retained its meaning. 

**Table 2:** Methods of assessment for technical capacities and participants.

Assessment method		Participants		Data to be collected	
Document review		N/A^a^		Policies that could support implementation of primary eye care (PEC)	
**In primary health care (PHC) facilities**					
	Structured questionnaire A		Heads of facilities, which can be any cadre		Facility practices that could support PEC implementation
	Structured questionnaire B		Community health extension workers		Health promotion practices that could support PEC implementation
	Observational checklist A		Heads of facilities		Equipment, consumables, infrastructure, and register data that could support PEC implementation in facilities
	Observational checklist B		Community health extension workers		Equipment, consumables, infrastructure, and register data that could support health promotion of PEC
**In purposively selected facilities**					
	In-depth interviews A		Heads of facilities		PHC experiences; extent to which PEC can be implemented in their facilities
	Structured questionnaire C		Village health workers		Perspectives on PEC promotion and prevention
**In each district**					
	In-depth interviews B		District PHC supervisors		PHC management experiences; extent to which PEC could be implemented in their districts

^a^N/A: not applicable; participants were not involved in the review of the document.

##### Study Area

**Figure 4:**
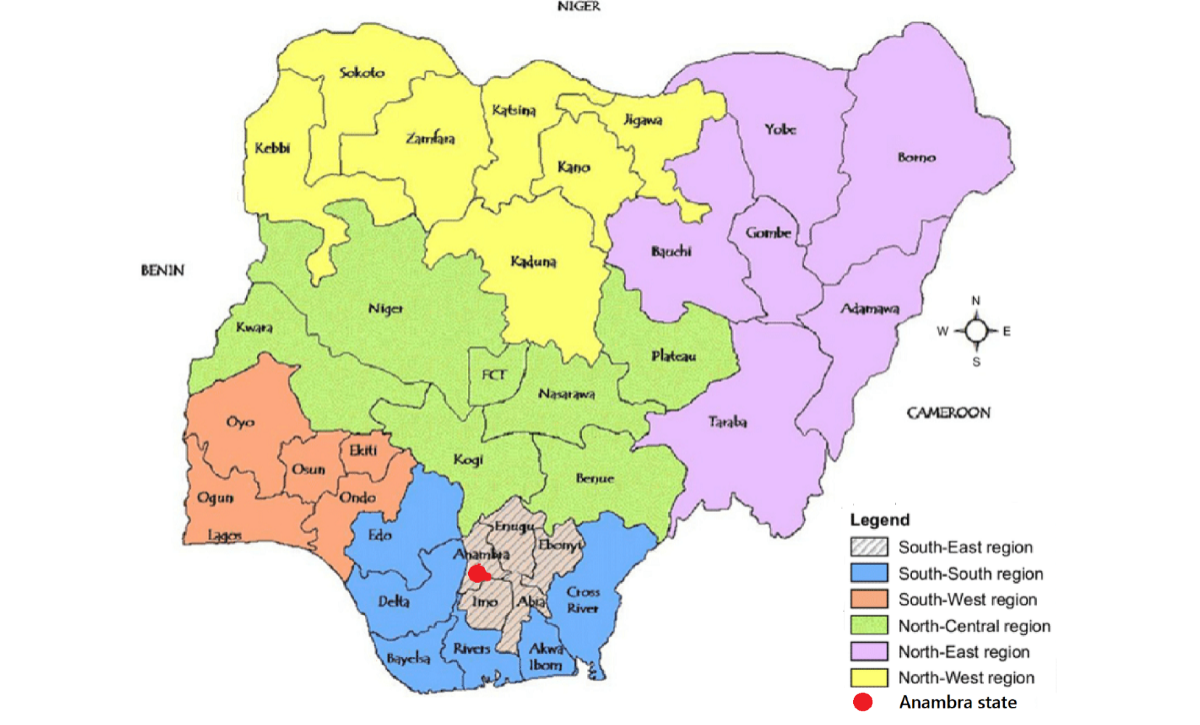
Map of Nigeria showing the six geopolitical zones and the 36 states [34].

Nigeria has 36 states in six geopolitical zones. Enugu State was selected for the pilot study and Anambra State for the main study, both of which are in the southeast zone (see Figure 4 [34]).

#### Pilot Study in Enugu State

A pilot study was undertaken in three PHC facilities in one district in Enugu State to assess all the data collection instruments. Appropriate amendments were made to the study tools based on the responses of participants.

#### Main Study in Anambra State

##### Overview

Anambra state has 21 local government areas, or districts, which can be stratified into urban, semiurban, and rural. The main occupations are agriculture, manufacturing, and commerce. The literacy rate of individuals aged 6 years and older is 75.1% [35], and 11.3% are considered to be poor [36].

##### Participants

Participants included heads of facilities, CHEWs, district supervisors, and VHWs. If a facility had two or more CHEWs, one was randomly selected. Data to be collected from each participant group are summarized in Table 2. Facility surveys were undertaken in primary health centers and health posts.

##### Selection of Districts, Facilities, and Participants

As this was a descriptive study, a sample of 48 facilities was estimated for a baseline study to be sufficient to determine a prevalence of 50% of PHCs with the technical capacity to implement PEC, with a margin of error of 20% with a 95% confidence level, a cluster design effect of 1.8, and accounting for a 10% nonresponse rate [37].

Facilities for inclusion in the main study in Anambra State, which has 21 districts with 235 PHC centers and 112 health posts (ie, a ratio of 2:1), were selected using a two-stage process. First, a list of districts was drawn up, stratified by rural, semiurban, and urban location, to create a sampling frame. Six districts were selected by selecting the appropriate number within each stratum to represent their distribution (ie, three semiurban to two rural to one urban) using simple random sampling. Second, within each district a list of PHC facilities was obtained from the National Primary Health Care Development Agency. The number of facilities—PHC centers and health posts—within each stratum was selected by probability proportionate to size in each district and to represent the 2:1 distribution of health centers and health posts.

The principal researcher administered the head-of-facility questionnaires and facility observational checklists; trained research assistants administered the CHEW and VHW questionnaires and the health promotion observational checklists. Paper forms were used to collect the data.

#### Preliminary Data Analysis and Purposive Selection of Participants for Qualitative Interviews

Interim data analysis was undertaken using predetermined criteria (eg, the availability of regular supervision, availability and use of standard operating procedures [SOPs], health workforce strength, and number of patients attending the facilities). The highest- and lowest-scoring facilities were stratified by location (ie, urban, rural, or semiurban) and type of facility (ie, health center or health post).

#### Structured Interviews With VHWs and Semistructured Interviews With Facility Heads and District Supervisors

Based on the preliminary analysis, nine facilities (ie, six health centers and three health posts) were purposively selected. The principal researcher conducted in-depth interviews with the heads of these facilities using semistructured topic guides. A total of 2 VHWs from each of the nine facilities were also randomly selected, and the trained research assistants administered structured questionnaires. Finally, the principal investigator conducted in-depth interviews with the district supervisors of each of the six districts using semistructured topic guides. All the interviews were conducted in English apart from the questionnaires for VHWs, which were administered in the local language by bilingual research assistants.

### Data Management

#### Overview

All the data from the two checklists and three questionnaires have been entered into specially prepared databases in Microsoft Access 2016 and transferred to Stata, version 15.1 (StataCorp LLC), using Stat/Transfer for analysis. Interviews with heads of facilities and district supervisors have been conducted and were recorded on an MP3 player. Verbatim transcription and reflection were ongoing during the interviews and evolving concepts were explored in subsequent interviews.

Quality assurance of data collection for the questionnaires was undertaken by training the research team with a daily debriefing. Each structured questionnaire was initialed by the research team member only when the form was correctly and completely filled out. For data entry, data validation rules were applied to the appropriate fields, which included range checks for numerical values. In addition, 10% of the questionnaires were randomly selected and data entry was cross-checked. During the semistructured interviews, the principal investigator was aware of her role as a benign interviewer and not a judgmental ophthalmologist. At the end of each interview, a summary of the participant’s views was read to them for confirmation. The interview recordings were transcribed by the principal investigator and checked for errors or omissions by replaying the tapes.

All data have been stored in a backed-up hard drive in a password-encrypted laptop and in the institution’s data repository (Filr) with controlled access limited to authorized users. Any data transferred through the internet have been encrypted. Data will be stored for 10 years to enable publications to be made from the data; they will then be deleted.

#### Data Analysis

##### Questionnaires and Checklists

Frequency tables will be generated from the data. Simple descriptive analyses will be performed (eg, the proportion of the facilities visited with tools for referrals). Existing capacities will be benchmarked against norms [38], when available (eg, staffing levels by cadre; SOPs; frequency of supervision; and some components of equipment, consumables, and medication, including systems to maintain the cold chain for vaccines). For indicators without norms, a descriptive analysis will be undertaken, benchmarking against the capacities required. The data will be analyzed based on the WHO health systems framework to highlight the elements that require strengthening.

##### In-Depth Interviews

Thematic analysis will be used to explore the data using OpenCode software, version 4.02. The data will be coded, categorized using the WHO health systems framework, and developed into themes. Data interpretation will be reviewed and discussed with the research team and qualitative experts. Final themes will be developed. The analysis will be supported by anonymized quotes from the data. Identification codes will be generated according to interview number, participant cadre, and type of facility. Reporting of the analysis of the interviews will follow COREQ (Consolidated Criteria for Reporting Qualitative Studies) guidelines [39].

#### Ethical Approval

Ethical approval was granted by the ethics review committees of the Federal Ministry of Health, Nigeria; the University of Nigeria Teaching Hospital; and the London School of Hygiene & Tropical Medicine. Written informed consent was obtained from each participant at the beginning of each interaction. For the interviews with heads of facilities and district supervisors, consent included permission to audio record the interviews and use anonymous quotes.

## Results

Consensus was reached among experts during the Delphi exercise regarding the technical complexity of the WHO-AFRO PEC package and the capacities needed to deliver it in PHC facilities. Based on the agreed-upon technical capacities, quantitative tools have been developed and relevant stakeholders have been identified to assess the technical capacity of PHC facilities to deliver the WHO-AFRO PEC package (ie, structured questionnaires, observation checklists, and topic guides of in-depth interviews).

Results from the pilot study highlighted large gaps in human resources for health and supervision at the community level, and the study tools were amended to accommodate this. The pilot study involved staff in three health centers in one district in Enugu State. All the data collection tools were pilot-tested apart from the topic guide for supervisors and the structured questionnaire for VHWs, as they were not available. Only one change was made to data collection, which was that the main survey questionnaire be administered to the appropriate cadre, as health promotion was mainly undertaken by a different cadre than anticipated.

Key findings were that none of the facilities had the full complement of staff, and none had a doctor or nurse midwife. The only in-service training that staff had received in the previous 12 months was in child health, maternal health, and HIV. Regarding health promotion, in each facility senior members of staff were providing health promotion in the community, which focused almost exclusively on maternal and child health. The only health promotion topic of relevance to eye care was not to self-medicate. The only form of transport provided to visit communities was a motorbike in one facility. No facility used forms for referral, which was done verbally. For facility-based management, all three were observed to have standing orders, and all reported supervision to be irregular. The main focus of the services delivered was maternal and child health, and none provided any eye care. Two facilities were able to test blood sugar, and none provided services for the elderly. In relation to equipment for eye care, one facility had a visual acuity chart, none had flashlights, and only one had antibiotic eye ointment. The facility survey in 48 PHC facilities has been completed as planned, and interviews with district supervisors and facility heads of the nine purposively selected facilities have been undertaken.

Capacities to deliver PEC are being analyzed, and gaps are being identified. Findings from all sources will be reviewed, including from the desk review of guidelines and policy documents. The convergence of findings will highlight whether gaps in the capacity to deliver PEC are due to limitations in the national guidelines or policies and/or whether they are due to limitations in the current delivery of PHC at district and/or facility levels.

## Discussion

This is the first study, to our knowledge, to assess the technical feasibility of integrating eye care into PHC in sub-Saharan Africa and the extent to which the health system needs to be strengthened to deliver it. An alternative approach to facilitate implementation in low-capacity settings would be to adapt the PEC package. There has been renewed interest in assessing the integration of services into health systems in LMICs [40], and the results from this study will be central to enabling policy makers to make an informed choice about what needs to be done to implement PEC in Nigeria.

The development of a conceptual framework for assessing health system interventions is important [41]. This study builds upon the technical complexity framework of Gericke to incorporate technical capacity assessments and will report a gap analysis in the system based on the WHO health systems framework. It is critical that countries implementing or scaling up new interventions have access to reliable, accurate, and comprehensive data on capacities and gaps in the system to deliver an equitable [42] and sustainable intervention. This study provides tools that could be adapted or modified for use in other countries in the region that plan to deliver the WHO-AFRO PEC package.

A limitation of the study is that it only addresses technical feasibility; other aspects of feasibility as delineated by Snowden, such as cultural, legal, financial, and political feasibility, may also need to be addressed. In addition, the assessment tools are cadre specific and designed for the Nigerian PHC context and may not be applicable to other settings. In this study, the sample size was 48 facilities. The study may not have been powered sufficiently to assess any capacity differences between health centers and health posts.

Results of the study will be disseminated to stakeholders in PHC and eye care in Nigeria by communique at stakeholders’ meetings and at local, national, and international ophthalmology and public health conferences, as well as in peer-reviewed journals.

## Abbreviations

CHEW: community health extension worker
COREQ: Consolidated Criteria for Reporting Qualitative Studies
LMICs: low- and middle-income countries
PEC: primary eye care
PHC: primary health care
SOP: standard operating procedure
VHW: village health worker
WHO: World Health Organization
WHO-AFRO: World Health Organization Regional Office for Africa
